# Cystic Arterial Disease Localized in the Media of the Popliteal Artery With Dissection

**DOI:** 10.7759/cureus.81263

**Published:** 2025-03-26

**Authors:** Akito Setoguchi, Saburo Kusumoto, Koji Hashizume, Junji Irie, Koji Maemura

**Affiliations:** 1 Department of Cardiovascular Medicine, Nagasaki Harbor Medical Center, Nagasaki, JPN; 2 Department of Cardiovascular Surgery, Nagasaki Harbor Medical Center, Nagasaki, JPN; 3 Department of Pathology, Nagasaki Harbor Medical Center, Nagasaki, JPN; 4 Department of Cardiovascular Medicine, Nagasaki University Graduate School of Biomedical Sciences, Nagasaki, JPN

**Keywords:** adventitial cystic disease, cystic arterial disease, dissection, media, popliteal artery

## Abstract

Adventitial cystic disease (ACD) of the popliteal artery is a rare disease that can cause intermittent claudication, and most cases show cysts located in the adventitia. We report a rare case with a cyst localized in the media and a dissection. The patient presented to the hospital with sudden-onset right lower limb pain. A lower limb ultrasound revealed an iso-echoic lesion in the right popliteal artery, leading to an initial diagnosis of thrombotic occlusion and subsequent thrombectomy. However, intravascular ultrasound suggested the lesion was outside the intima, and magnetic resonance imaging findings indicated ACD. Consequently, cyst resection with expanded polytetrafluoroethylene (ePTFE) graft reconstruction was performed. Histopathological examination revealed dissection associated with cystic medial necrosis. However, the condition was diagnosed as cystic arterial disease due to its similarity to previously reported cases and the inadequacy of the term "adventitial" cystic disease.

## Introduction

Adventitial cystic disease (ACD) is an uncommon pathology in which cysts form within the adventitia of arteries and veins, potentially leading to compression of the lumen and subsequent circulatory disorders. More than 700 cases have been reported, with approximately 80-85% involving the popliteal artery. Other reports have described cases in the external iliac, common femoral, radial, ulnar, and brachial arteries [1]. Rare cases have been reported in which lesions arise in the media [2-11]. Further, the etiology of ACD has not been determined, and whether treatment should be the same as for cysts located in the media remains controversial. Here, we present a case of cystic arterial disease localized in the media of the popliteal artery with dissection.

## Case presentation

An 82-year-old man experienced sudden pain in the right lower limb while walking and visited our hospital the next day because the pain persisted. The patient experienced severe pain even at rest but had no motor or sensory deficits. He had never experienced similar symptoms before. He had no history of trauma but exhibited hypertension, hyperlipidemia, and type 2 diabetes. Physical examination on admission revealed blood pressure of 179/93 mmHg, heart rate of 79 beats/min and regular, body temperature of 36.7°C, respiratory rate of 16 breaths/min, and body mass index of 25.3 kg/m2. The right lower leg was cold, and no pulses were palpable in the right posterior tibial or dorsal pedis arteries. Laboratory data revealed normal levels of both creatine kinase (126 U/L; reference range = 41-153 U/L) and C-reactive protein (0.13 mg/dL; reference range = 0.00-0.14 mg/dL).

**Figure 1:**
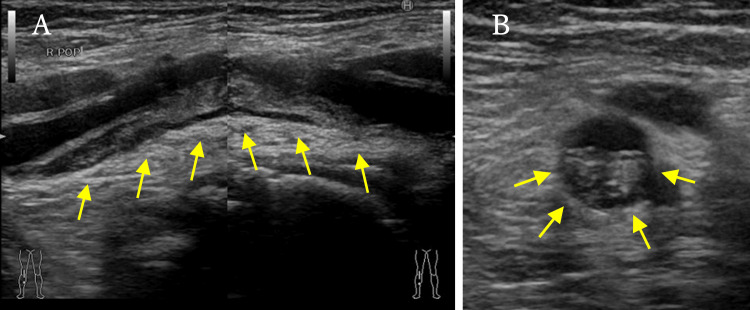
Ultrasonography of arteries in the lower extremity. (A) An iso-echoic lesion is located in the right popliteal fossa (arrows). (B) The lesion narrowed the lumen of the blood vessel (arrows).

Ultrasonography of arteries in the lower extremity revealed an iso-echoic lesion in the right popliteal fossa (Figure 1).

**Figure 2:**
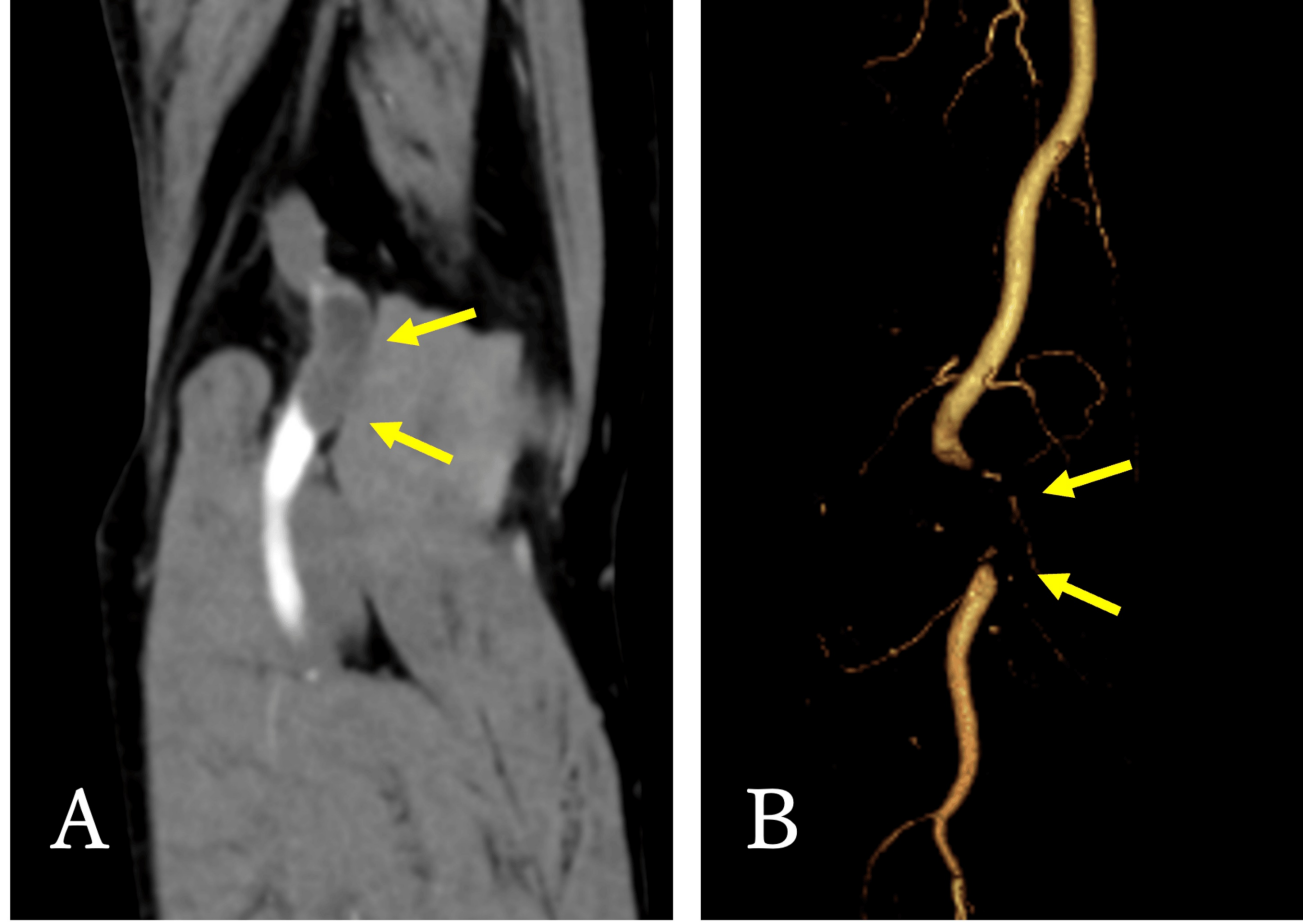
Contrast-enhanced computed tomography. (A) A low-density area was apparent in the right popliteal fossa (arrows). (B) The popliteal artery was almost occluded (arrows).

Contrast-enhanced computed tomography revealed a low-density area in the right popliteal artery but preserved arterial flow below the knee (Figure 2).

**Figure 3:**
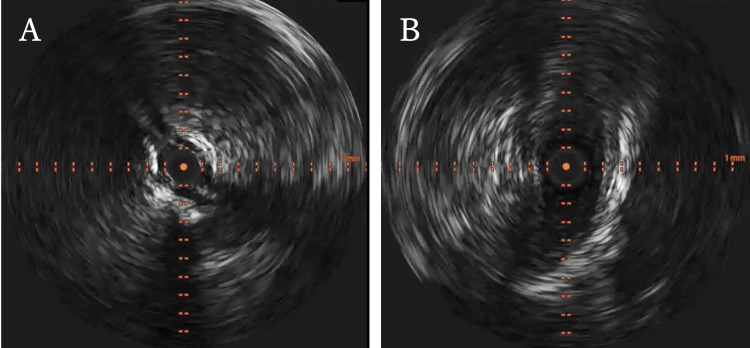
Intravascular ultrasound (IVUS) before and after treatment with Fogarty catheter. (A) IVUS revealed an iso-echoic lesion at the right popliteal artery. (B) Repeat IVUS revealed enlargement of the vascular lumen, with the lesion located outside the intima.

We suspected thrombotic occlusion in the right popliteal artery below the knee, and thrombectomy was performed the same day. Arteriography revealed complete occlusion of the right popliteal artery. Intravascular ultrasonography (IVUS) revealed an iso-echoic lesion at the same site (Figure 3A).

A Fogarty catheter was used once, but no thrombus could be retrieved. Repeat IVUS revealed enlargement of the vascular lumen, with the lesion located outside the intima (Figure 3B). After thrombectomy using the Fogarty catheter, both the right posterior tibial and dorsalis pedis pulses became palpable. Ankle-brachial index on the right leg was 1.08.

**Figure 4:**
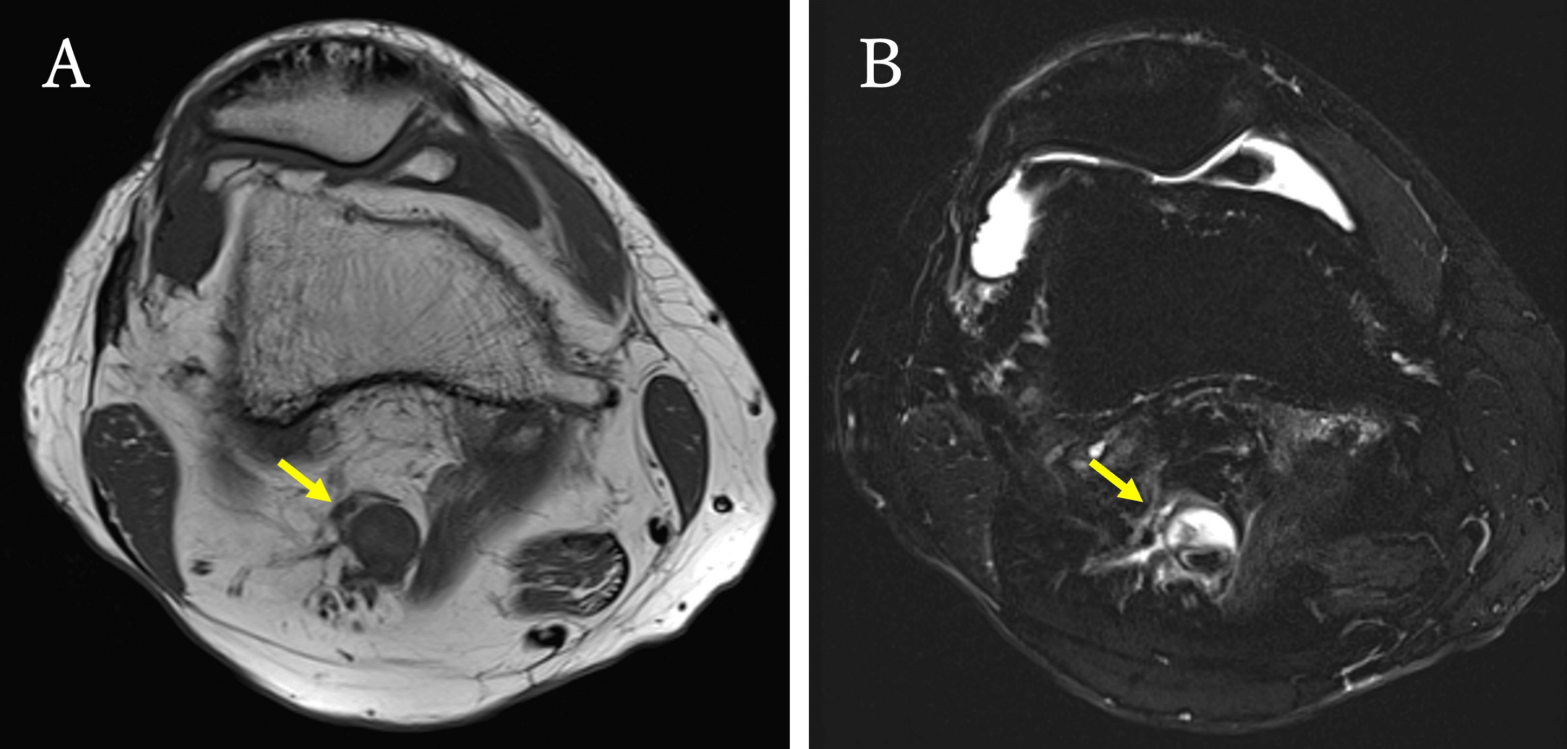
Magnetic resonance imaging. (A) T1-weighted imaging shows a low-intensity area in the right popliteal artery (arrow). (B) T2-weighted imaging shows a high-intensity area in the same region of the right popliteal artery (arrow).

Axial magnetic resonance imaging (MRI) was performed for further investigation, revealing a cystic lesion in the popliteal artery (Figure 4).

**Figure 5:**
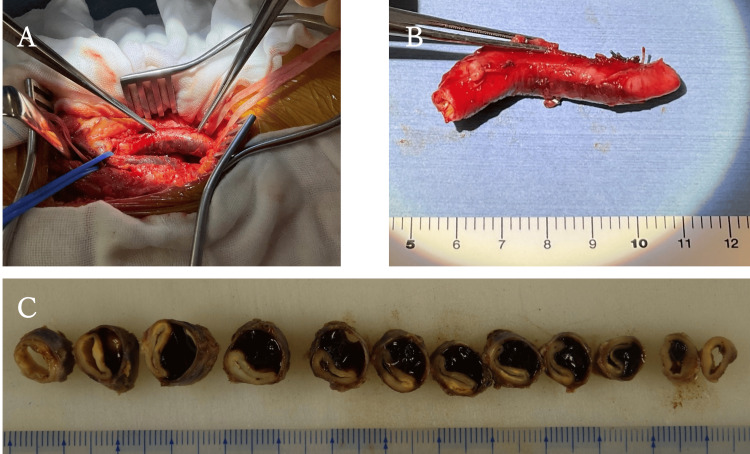
Intraoperative findings and macroscopic appearance of the specimen. (A) Intraoperative findings show a dark-red lesion in the wall of the popliteal artery. (B) Surgical specimen of the right popliteal artery. (C) Cross-section of the artery shows a collapsed artery and a cyst with mucoid material.

Based on these findings, ACD was diagnosed. We decided on surgical treatment, and a cardiovascular surgeon performed cyst resection and synthetic graft reconstruction with expanded polytetrafluoroethylene (ePTFE) graft (Figure 5A).

Macroscopic examination of the resected popliteal artery showed no cystic lesions in the adventitia (Figure 5B). A cross-section of the artery showed a collapsed artery and cyst with mucoid material (Figure 5C).

**Figure 6:**
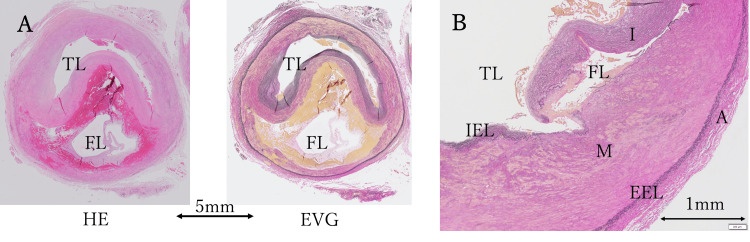
Pathological findings. (A) Cross-section of the popliteal artery demonstrates a true lumen (TL) and a false lumen (FL) (hematoxylin and eosin (HE) stain, ×20). The TL contains erythrocytes, while the FL shows erythrocytes and mucoid material (Elastica van Gieson (EVG) stain, ×20). (B) This section shows an intimal tear, suggesting blood inflow into the FL. Note the intimal thickening (EVG stain, ×40). TL: true lumen; FL: false lumen; I: intima; M: media; A: adventitia; IEL: internal elastic lamina; EEL: external elastic lamina.

One cross-section of the popliteal artery demonstrated a cyst containing erythrocytes and mucoid material, with the mucoid material located only in the media of the popliteal artery (Figure 6A).

**Figure 7:**
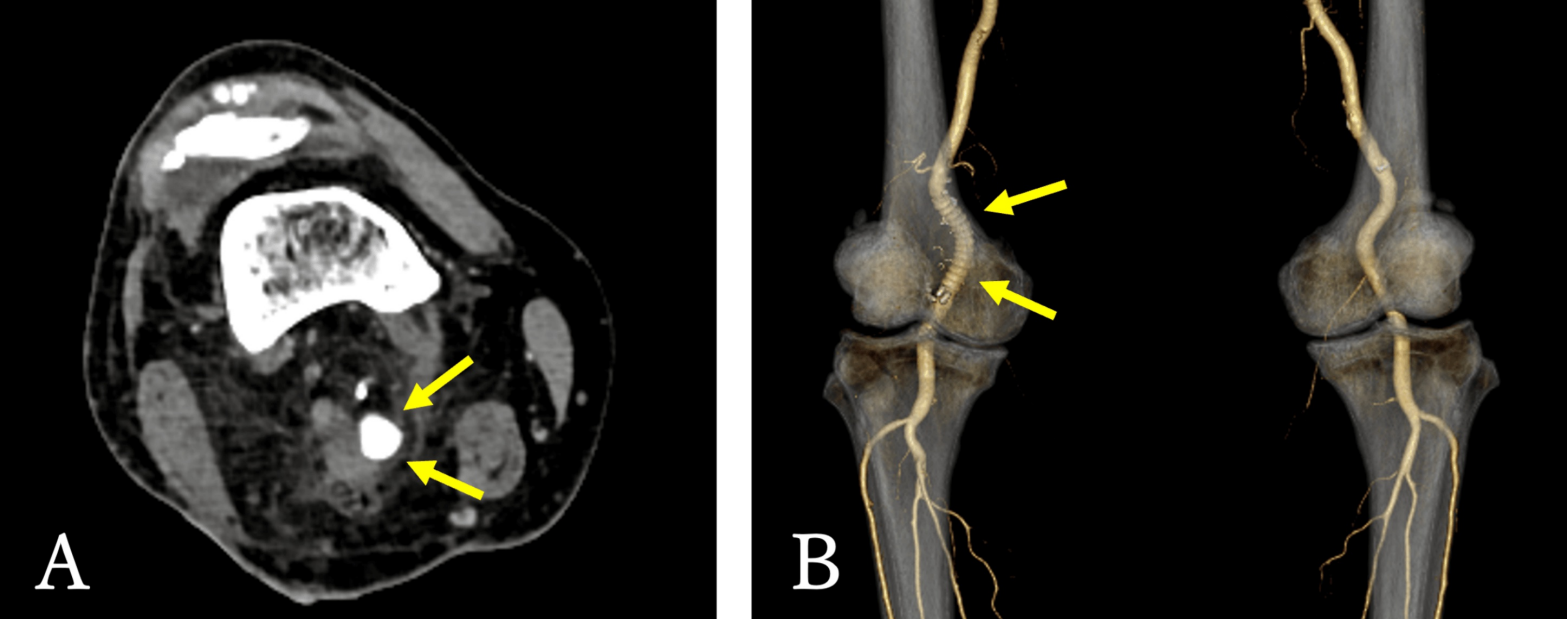
Postoperative contrast-enhanced computed tomography. (A) The right popliteal artery was replaced with an expanded polytetrafluoroethylene (ePTFE) graft (arrows). (B) Improved blood flow in the right lower limb (arrows).

Another section showed intimal thickening and tearing (Figure 6B). These findings suggested blood inflow into a false lumen. Postoperatively, blood flow in the right lower limb has improved (Figure 7), and the patient continues to take apixaban 10 mg/day.

As of six months postoperatively, the patient was able to walk without difficulty. To prevent complications, squatting was discouraged. Follow-up visits are scheduled annually.

## Discussion

Atkins and Key were the first to document ACD in 1947 in London [12]. This unusual non-atherosclerotic condition affects peripheral vessels, comprising a cystic tumor of blood vessels with accumulation of mucinous fluid inside the adventitia. When the illness starts to manifest, symptoms such as claudication and swelling of the limb appear when vessel compression occurs.

The etiological factors remain poorly understood. Four main theories have been proposed for the etiology of ACD: repetitive trauma theory, systemic disorder theory, developmental theory, and articular/synovial theory [13,14]. The repetitive trauma theory suggests that repeated flexion and extension of the joint induce chronic damage in the popliteal artery, causing adventitial cystic degeneration. However, this theory has been called into question by the fact that only about 4% of patients have a history of trauma [1]. In addition, some reports have involved young patients or early recurrence, which would not be readily explained by repeated trauma, so this theory is not well supported [15,16].

The systemic disorder theory postulates that a general connective tissue disorder causes degeneration and cyst formation in the adventitia. However, since a few case reports have described systemic disease as well as traumatic injuries, the association is thought to be weak.

The developmental theory suggests that mesenchymal mucin-secreting cells that generally form the knee joint are incorporated into the adventitia of the vessels during development. However, this theory has difficulty explaining recurrence after cyst removal or within the graft [17,18].

Finally, the articular/synovial theory proposes a connection between the adventitial cyst and the adjacent knee joint capsule. In such cases, ductus communication between the cyst and articular capsule can be recognized on diagnostic MRI and intraoperatively. In a previous study of 729 cysts, 122 (17%) were identified as showing connections to joints [1]. Further, all reported cases of adventitial cysts have involved lesions around joints, supporting this theory.

In the present case, the cyst was in the media, not the adventitia. Only 10 other cases of ACD localized in the tunica media have been reported (Table 1).

**Table 1:** Cases of cystic artery disease located in the media.

References	Journal	Publication year	Age	Sex	Lesion site	Ultrasonography	Treatment	Outcome
Andersson et al. [2]	Acta Radiol	1959	48	Male	Left popliteal artery	-	Cyst resection and reconstruction with arterial transplant	No symptoms for 18 months
Powis et al. [3]	Surgery	1970	35	Male	Left popliteal artery	-	Cyst incision and decompression	Reoperation after 1 year
Terry et al. [4]	Hum Pathol	1981	42	Male	Left popliteal artery	-	Cyst resection and vein graft reconstruction	Reoperation twice
Inoue et al. [5]	Ann Vasc Surg	1992	72	Male	Left popliteal artery	Low echo	Cyst resection and vein graft reconstruction	No symptoms for 2 years
Noda et al. [6]	J Jpn Surg Assoc	1992	56	Male	Left popliteal artery	-	Cyst resection and vein graft reconstruction	-
Akiyama et al. [7]	J Jpn Surg Assoc	1998	55	Male	Right popliteal artery	Water density	Cyst resection and vein graft reconstruction	-
Unno et al. [8]	Surg Today	2000	40	Male	Left popliteal artery	-	Cyst resection and vein graft reconstruction	-
Kobayashi et al. [9]	Okayama R C Hosp J Med	2001	49	Male	Right popliteal artery	-	Cyst resection and synthetic graft reconstruction	-
Yusa et al. [10]	Jpn J Vasc Surg	2004	59	Male	Left popliteal artery	Low echo	Cyst resection and vein graft reconstruction	-
Yoshino et al. [11]	Ann Vasc Dis	2019	65	Female	Right popliteal artery	-	Cyst resection and vein graft reconstruction	No symptoms for 2 years
This case	Cureus	2025	82	Male	Right popliteal artery	Iso echo	Cyst resection and synthetic graft reconstruction	No symptoms for 6 months

The mean age for these 11 cases was 55 years (interquartile range: 42-65 years), and only one case was female. All cases sought medical attention due to intermittent claudication, with symptoms either gradually worsening over several months or, as in this case, presenting suddenly. The lesions were located in the popliteal artery in all cases, and cyst resection was selected in all but one case. That patient without cyst resection showed recurrence one year after cyst incision. Pathologically, communication with the joint cavity was observed in one case. On the other hand, the present patient had no history of systemic connective tissue disease, repetitive trauma, or even daily exercise. In addition, no connection was seen between the medial cyst and the adjacent knee joint on preoperative imaging, intraoperative findings, or histopathological examination. A characteristic finding in this case was that the preoperative ultrasonography and IVUS showed an iso-echoic lesion. In contrast, all previously reported cases with documented ultrasound findings exhibited hypoechoic lesions. This iso-echoic appearance was due to blood inflow caused by dissection, which was considered the reason for the rapid worsening of symptoms.

Although few cases of ACD complicated by dissection or intimal tear have been reported, there are cases that presented pathologically with subintimal dissection and similar findings on ultrasonography [19].

In the present case, a Fogarty catheter was used before surgery, but the ultrasound findings were from before thrombus removal, suggesting that the dissection had been present before thrombus removal.

Interestingly, no reports have described cystic medial necrosis originating from the popliteal artery. Cystic medial necrosis is a degenerative disease characterized by a loss of smooth muscle cells from the vascular media, fragmentation of elastic fibers, and deposition of acidic mucopolysaccharides. This pathology occurs in 8-19% of cases of aortic dissection, and acquired factors such as aging and high blood pressure are considered responsible [20].

Based on the pathological findings, the present case was initially considered to represent cystic medial necrosis. The false lumen contained a mixture of mucoid materials and blood, suggesting that the entrance of the medial false lumen was an intimal tear. We think that degeneration of the popliteal artery occurred, and an intimal tear following atherosclerosis of the intima allowed the entry of blood into the false lumen. However, as previously mentioned, there have been no reported cases of cystic medial necrosis in the popliteal artery. Furthermore, ACD localized in the media has also been suggested to have medial degeneration as a potential etiology [2,5,7,10], making it difficult to distinguish it from cystic medial necrosis completely. Because distinguishing between these conditions using preoperative imaging (ultrasound, CT, and MRI) is difficult, cystic arterial disease occurring in the media is treated the same as ACD. However, some aspects of the disease may have different etiologies. Therefore, we propose a diagnosis of cystic arterial disease or cystic medial degeneration instead of ACD.

## Conclusions

We have presented a rare case of cystic arterial disease localized in the media with subintimal dissection, successfully treated with surgical repair. Pathologically, the lesion was considered a popliteal artery dissection associated with cystic medial necrosis. However, given its similarity to previously reported cases with cysts in the media, we diagnosed it as cystic arterial disease rather than "adventitial" cystic disease.

Cystic arterial disease involving lesions in the media is extremely rare and has been treated in the same manner as ACD, but further study is warranted to determine the optimal treatment and long-term prognosis. The etiology of this condition is unknown but may involve a combination of several clinical factors.

## References

[1] Desy NM, Spinner RJ (2014). The etiology and management of cystic adventitial disease. J Vasc Surg.

[2] Andersson T, Gothman B, Lindberg K (1959). Mucinous cystic dissecting intramural degeneration of the popliteal artery. Acta Radiol (Stockh).

[3] Powis SJ, Morrissey DM, Jones EL (1970). Cystic degeneration of the popliteal artery. Surgery.

[4] Terry JD, Schenken JR, Lohff MR, Neis DD (1981). Cystic adventitial disease. Hum Pathol.

[5] Inoue Y, Iwai T, Ohashi K (1992). A case of popliteal cystic degeneration with pathological considerations. Ann Vasc Surg.

[6] Noda H, Fujioka K, Zempo N (1992). Cystic adventitial disease of the popliteal artery: a case report and summary of Japanese literature. J Jpn Surg Assoc.

[7] Akiyama Y, Aoyama H, Motegi K, Ishitobi K (1998). Two cases of adventitial disease of the popliteal artery - with reference to the pathology and selection of operative procedure. J Jpn Surg Assoc.

[8] Unno N, Kaneko H, Uchiyama T, Yamamoto N, Nakamura S (2000). Cystic adventitial disease of the popliteal artery: elongation into the media of the popliteal artery and communication with the knee joint capsule: report of a case. Surg Today.

[9] Kobayashi H, Hashimura S, Mifune H (2001). A case of cystic adventitial disease of popliteal artery. (Article in Japanese). Okayama R C Hosp J Med.

[10] Yusa Y, Inoue Y, Igari T, Kanno N, Koike M, Iwai T (2004). A case of spontaneous exaggeration in cystic adventitial degeneration of the popliteal artery. (Article in Japanese). Jpn J Vasc Surg.

[11] Yoshino S, Inoue K, Yoshiya K (2019). Cystic arterial disease located only in the media of the popliteal artery: a case report. Ann Vasc Dis.

[12] Atkins HJ, Key JA (1947). A case of myxomatous tumour arising in the adventitia of the left external iliac artery. Br J Surg.

[13] Tsolakis IA, Walvatne CS, Caldwell MD (1998). Cystic adventitial disease of the popliteal artery: diagnosis and treatment. Eur J Vasc Endovasc Surg.

[14] Flanigan DP, Burnham SJ, Goodreau JJ, Bergan JJ (1979). Summary of cases of adventitial cystic disease of the popliteal artery. Ann Surg.

[15] Jones DW, Rezayat C, Winchester P, Karwowski JK (2012). Adventitial cystic disease of the femoral vein in a 5-year-old boy mimicking deep venous thrombosis. J Vasc Surg.

[16] Patel SM, Patil VA, Pamoukian VN (2008). Interposition grafting of popliteal artery cystic adventitial disease: case report. Vasc Endovascular Surg.

[17] Maged IM, Turba UC, Housseini AM, Kern JA, Kron IL, Hagspiel KD (2010). High spatial resolution magnetic resonance imaging of cystic adventitial disease of the popliteal artery. J Vasc Surg.

[18] Aerden D, Van Nieuwenhove Y, Debing E, Van den Brande P (2000). Perivascular cystic degeneration of a saphenous vein graft. Eur J Vasc Endovasc Surg.

[19] Fitzjohn TP, White FE, Loose HW, Proud G (1986). Computed tomography and sonography of cystic adventitial disease. Br J Radiol.

[20] Larson EW, Edwards WD (1984). Risk factors for aortic dissection: a necropsy study of 161 cases. Am J Cardiol.

